# Physiologically based pharmacokinetic modeling of cefotaxime to inform pediatric dosing in renal impairment

**DOI:** 10.1007/s00228-026-04121-8

**Published:** 2026-07-10

**Authors:** Najia Rahim, Muhammad Sarfraz, Muhammad Wahajuddin

**Affiliations:** 1 Department of Pharmacy Practice, Dow College of Pharmacy, Dow University of Health Sciences, Karachi, Sindh Pakistan; 2 College of Pharmacy, Al Ain University, Al Ain, Abu Dhabi United Arab Emirates; 3 Institute of Cancer Therapeutics, School of Pharmacy & Medical Sciences, Faculty of Life Sciences, University of Bradford, Bradford, West Yorkshire United Kingdom

**Keywords:** Cefotaxime, Dose recommendations, Renal impairment, PBPK modeling

## Abstract

**Background:**

Cefotaxime (CFT) is a broad spectrum, third-generation cephalosporin antibiotic prescribed for the treatment of severe infections, yet dosing guidelines for pediatric with renal impairment is scarce. The current study aimed to develop a physiological based pharmacokinetic (PBPK) model to characterize CFT disposition and model-based recommend dose adjustments in pediatrics with renal impairment.

**Methods:**

Initially, the PBPK model of CFT was developed in adults with normal renal function before being scaled to pediatrics, considering age-related physiological changes using GastroPlus® software. Renal impairment was modelled through optimization of tubular secretion and glomerular filtration parameters based on observed data for both adults and pediatric populations.

**Results:**

The model reasonably reproduced the observed pharmacokinetic profiles and showed acceptable agreement with the data (fold error ranges 0.84–1.37) or with renal impairment (fold error ranges 0.87–1.12). When compared to children with normal renal function, the predicted AUC0−∞ in children with renal impairment increased to 1.22- and 1.89-fold for moderate and severe renal impairment, respectively. Model-informed dose recommendations were 60% and 48% of the standard pediatric dose for moderate and severe renal impairment, respectively.

**Conclusion:**

This PBPK framework supports rational, model-informed dosing of CFT in pediatric patients with varying renal impairment and supports dose recommendation development for high-risk populations.

**Graphical abstract:**

**Figure:**
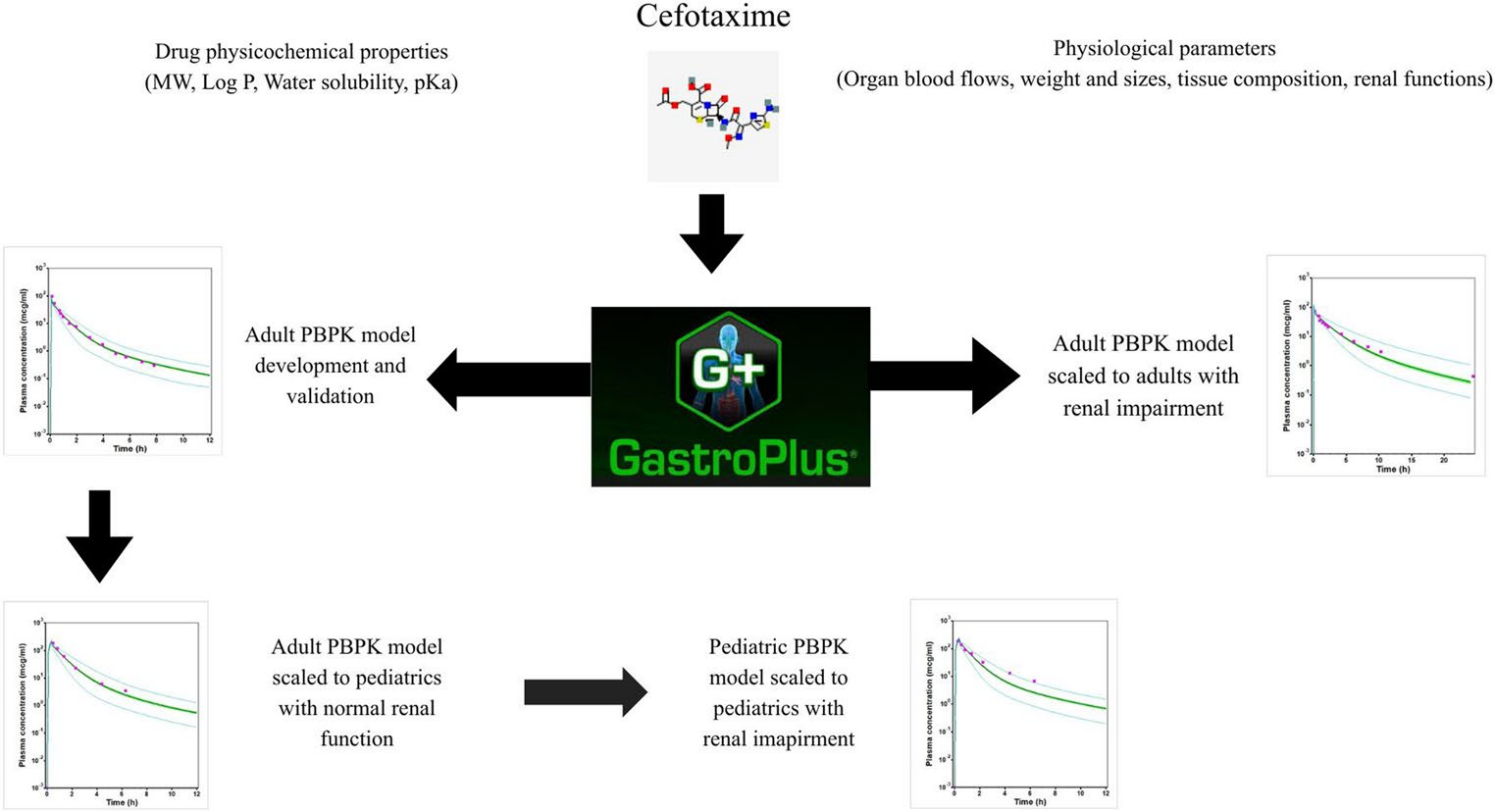

**Supplementary Information:**

The online version contains supplementary material available at 10.1007/s00228-026-04121-8.

## Introduction

Cefotaxime (CFT) is a third-generation cephalosporin administered for infections caused by a wide range of Gram-positive and Gram-negative bacteria. It is prescribed for the treatment of lower respiratory tract, central nervous system, genitourinary tract, bone and joint, intra-abdominal, and skin infections. CFT is recommended for use in children (dose range: 50–200 mg/kg/day body weight (BW) for children below 12 years). Adults and children above 12 years have same dose range (dose range: 1–2 g/day) via intravenous infusion (IV) or intramuscular injection [40]. CFT exhibited linear pharmacokinetics (PK) with the elimination half-life of 1–1.5 h, with extensive distribution in blood and extracellular fluids, and penetration to the cerebrospinal fluid (CSF) under inflammatory conditions. Elimination is primarily renal, with 50–60% of the administered dose eliminated unchanged in the urine, and 15–20% excreted as the only known active metabolite (desacetyl cefotaxime) by Carboxylesterase 1 (CES1) [26].

Renal elimination of CFT is via glomerular filtration and active secretion by organic anion transporters (OATs) that are located at the basolateral membrane of the proximal tubule in the kidney. Previous studies have shown that CFT exhibits higher affinity for OAT3 compared with OAT1 and OAT4, suggesting transporter-mediated clearance as a major determinant of internal exposure [50]. Consequently, physiological changes associated with renal impairment (RI) influences the dosing of CFT in adults and children, which is calculated based on creatinine clearance (Cl_cr_) [22, 33]. This is because RI leads to decreased clearance (CL), likely driven by reduced transporter activity (OAT1/3), leading to increased CFT internal exposure and accumulation [47]. It is recommended to reduce the CFT dose in patients suffering from severe renal impairment (RI) based on creatinine clearance to avoid drug accumulation [6]. However, specific protocols remain unidentified (Drug.Com) [7]. Furthermore, pediatric-specific dosing guidance remains sparse, and standardized adjustment strategies are lacking [11].

From a pharmacodynamic perspective, CFT displays time-dependent antibacterial activity. Therefore, the value of % T > MIC i.e. the percentage of time that passes between subsequent antibiotic treatments while the antibiotic’s concentration remains above the minimum inhibitory concentration (MIC) is very crucial. The MIC of CFT for susceptible, intermediate susceptible, and resistant causative bacteria (anaerobic bacteria) ranges from 1 mg/L to 4 mg/L [10]. Another study reported a MIC range of 0.016-2.0 mcg/ml for CFT [45]. Antibacterial activity of CFT was achieved when the % T > MIC reached 50% and 80% of the dosing interval (i.e., 24 h) for *S. pyogenes* and *E. coli*, respectively [16]. Therefore, the pharmacological activity for the adjusted doses should be evaluated.

Extrapolation of adult dosing regimen to pediatrics are commonly based on the age-appropriate and weight-based scaling [2]. The developmental changes and maturation processes that impact drug pharmacokinetic (PK) and pharmacodynamics (PD) complicate this process [28, 32]. Clinical PK data in children with RI is extremely limited, with only one clinical study reporting changes in CFT’s internal exposure in children with RI.Paap et al., [34] The study suggested adjusting the doses in children with RI, as the clearance of CFT was found to be reduced in moderate and severe RI [34].

Physiologically based pharmacokinetic (PBPK) models can simulate drug disposition in special populations, which can help with decision-making for selecting dosages [13, 43]. By mechanistically representing the size, constitution, and blood flow of different tissues and organs, PBPK models capture age-associated changes in anatomy and physiology based on the available data. This framework is especially helpful in pediatrics for comprehending the change in CL among pediatrics patients, where limited clinical data is available. Using PBPK models, the use of drugs in pediatric patients has been studied and rationalized [20, 37].

Few PBPK models of CFT were reported for adults with RI and for preterm/term neonates using PK-Sim^®^ [27, 51]. A validated PBPK model addressing the pediatric population with impaired renal function has not yet been reported. Hence, the current study aimed to develop a PBPK model of CFT using GastroPlus^®^. The PK of CFT in children with varying renal functions was predicted using this PBPK model, and the model was also utilized to recommend a dose considering the simulation results. The study assesses RI-related changes in active secretion for OAT1/3 substrates, improving confidence in prospective predictions of PK and supporting model-informed dose adjustments for OAT1/3 substrates in populations with RI, especially pediatrics.

## Methods

A systematic literature search was executed via PubMed, Science Direct, Google Scholar, and Cochrane to find clinical PK studies describing the systemic concentration–time profiles of CFT in humans. Fourteen studies were found suitable for model development and validation, including eleven studies in adults and three in pediatrics. Retrieved studies were examined to collect relevant information on dosage schedules, PK parameters, PK time-course profiles, and demographic characteristics (health status, gender, age, weight, body mass index (BMI), and height). Graph Digitizer^®^ (version 2.26) was utilized to retrieve the experimental data points from the plasma concentration–time profiles. Table S1-S3 list the characteristics of clinical studies that were used to develop and validate the PBPK model.

### Modeling software

The GastroPlus^®^ software version 9.9 (Simulations Plus Inc., Lancaster, CA, USA) was used for the development of the PBPK model, which offers a user-friendly interface with proficiency in mathematical modelling. Age-dependent physiological and anatomical data for a variety of species are built into the software’s database, which helps to refine model input parameters.

### PBPK model development

Model construction was based on drug-dependent variables, system-specific characteristics, and clinical trial inputs. Clinical study data’s demographics were used to generate virtual subjects (Tables S1-S3). GastroPlus^®^’s population databases were used to supplement any missing data on age, weight, height, or BMI (body mass index).

CFT is characterized as a hydrophilic weak acid with a molecular weight of 455.47 g/mol and has dissociation constants of pKa and pKb of 2.51 and 3.98, respectively. Desacetyl CFT is the main metabolite produced by the cleavage of the parent molecule, and CFT undergoes little metabolism [48].

The initial PBPK model was developed with all default parameters with non-mechanistic clearance using PBPK module of GastroPlus^®^. The predefined default values of GastroPlus^®^ software were used to create a standard virtual individual with a European population (age = 25 years, weight = 69 kg, height = 176 cm, BMI = 22.19 kg/m^2^), which matched with the clinical study utilized for the initial model development [31]. The observed data points from the plasma concentration profiles of the selected study were retrieved and loaded as ipd. files [31]. CFT distribution in different tissues was initially described as a perfusion-limited model. After running simulations, this distribution method was discontinued as it was unable to simulate observed data and replaced with the permeability-limited method. The justification is that CFT is a Biopharmaceutics Classification System (BCS) class III characterized by low permeability. It’s a relatively hydrophilic drug with a low volume of distribution and higher concentration in blood and extracellular fluids, further supporting the use of a permeability-limited model to describe its distribution more accurately [3]. The permeability-surface-area product (PStc) was incorporated to characterize drug distribution across individual organs. PStc value was optimized utilizing observed data to simulate CFT exposure. Furthermore, we modified the fraction unbound in plasma (fup) to improve the prediction accuracy. Therefore, fup was optimized from 42.26% to 60%, consistent with reported protein binding ranges (35–45%) [9, 14] (Table S4 and Table S5).

Next, the OAT3 transporter was added to incorporate mechanistic clearance in place of non-mechanistic clearance. Michaelis–Menten parameters (K_m_ and V_max_) for OAT3 extracted from a previous study were included in the initial model.(Yee, et al.,2013) Renal transporter activity on the basolateral membrane was modified depending on plasma concentration-time profiles to optimize active renal secretion, assuming that passive permeability was minimal. Optimized V_max_ values for the transporter in order to reproduce the observed plasma-drug concentration profiles are mentioned in Table S5.

There are a few reports suggesting that CFT is a substrate of CES1; however, these reports lack the enzyme kinetics [39]. Consequently, CES1-mediated metabolism was not included in the model, and non-renal clearance was added instead. Likewise, some reports suggested that CFT is a Pg-P substrate; this transporter was also excluded from the model due to insufficient quantitative data [38]. The accuracy of the model’s predictions was evaluated by superimposing the observed data on predicted concentration–time curves [46].

### PBPK model validation

The developed PBPK model’s performance was assessed using a goodness of fit analysis in addition to the visual inspection of graphical presentations [12]. It was accomplished by comparing the observed data from reported clinical studies with the predicted PK profile of CFT. The observed values of PK parameters (the maximum plasma concentration, C_max_, the plasma drug concentration-time curve from zero to the last time point, AUC_0−t_, and the area under the plasma drug concentration-time curve from zero to infinity, AUC_0−∞_) were compared to the predicted values. Arithmetic mean of PK parameters values were used to calculate fold error (FE). Equation (1) was used to calculate the predicted versus observed FE.

1 $$Fold\;Error\;\left(FE\right)=\frac{Predicted\;PK\;Parameter}{Observed\;PK\;Parameter}$$

The population simulator of GastroPlus^®^ was utilized to get the predicted AUC_0−∞_ mean values from the 95th percentiles using Monte Carlo simulations. The virtual adult populations in GastroPlus^®^ were used to develop and validate the model. Plasma concentration-time profiles were generated for different dosages ranging from 500 to 2000 mg administered to the adult populations to evaluate the prediction accuracy of the adult model [9, 14, 15, 17, 18, 21, 25, 31, 42].

### Configuring the PBPK model in adults with renal impairment

PBPK modeling is a useful approach for dose recommendations for patients with varying degree of RI. The US Kidney Disease Outcomes Quality Initiatives (KDOQI) classify the severity of RI into five stages: normal, mild, moderate, severe, and end-stage [19]. The renal clearance (CL_renal_) of a drug can be directly impacted by pathophysiological changes brought on by such disease, including increased plasma protein (albumin), stomach emptying, haematocrit, and intestinal transit time. The initial PBPK model was modified for patients with RI by incorporating a scaling factor of OAT3 V_max_, reducing glomerular filtration rate (GFR), and evaluating it using observed PK data [22].

For this scaling of transporter kinetics, the Dettli method and Intact Nephron hypothesis were used [4, 5]. The scaling factor was determined on the basis of the deterioration in renal function as well as GFR in RI patients (Table S6). A similar approach has been used by other researchers to develop models for various drugs in individuals with RI [1, 35]. The model applicability in patients with RI receiving different doses of CFT was verified using data from two clinical studies [22, 33].

### Modification of dosage for adults with renal impairment

PBPK model for adults with moderate, severe, and end-stage RI was modeled for the dose modification. The ratios of AUC_0−∞_ between patients with RI and healthy individuals were compared across renal function categories (normal renal function, moderate impairment, severe impairment, and end-stage renal disease).

### Model scaling to pediatrics

The qualified adult PBPK model was scaled to pediatric population. The GastroPlus^®^ preset age-related physiological changes, such as blood flow to different organs and organ sizes, were taken into account for this purpose. The activity of the renal transporter (OAT3) and GFR were scaled to pediatric population using a scaling factor. Three clinical studies involving pediatric subjects (age range 7 months to 16 years) were utilized. The ratio of renal clearance in children to that in adults was used to calculate the scaling factor (Table S7). Other researchers also used this strategy for scaling of adult models to pediatric populations [8, 35]

### Model scaled to pediatrics with renal impairment

PBPK model in pediatrics without RI was scaled to pediatric patients with RI using the observed data from previous study involving pediatrics of the ages 7 to 16 years. Paap et al., [34] For initial model scaling to pediatrics with RI, we utilized the PEAR physiology module of GastroPlus^®^ and generated one virtual subject reflecting the demographic average of the pediatric population was constructed. The subject was 12 years old, weighed 39 kg, and had moderate and severe RI. V_max_ of OAT3 was scaled to generate different stages of RI (Table S8), as we did in PBPK model scaling to adults with RI. Simulation results were evaluated for the model precision and applicability using Monte Carlo simulation (*n* = 100 virtual subjects).

### Modification of dosage in pediatrics with renal impairment

Model based dosage recommendations for pediatric patients with RI were accomplished by utilizing the PBPK models of pediatrics with RI. CFT exposure in RI can be predicted using AUC_0−∞_ increase. Pediatrics patient’s CFT PK profiles were predicted using simulations, and these profiles were compared to those of children with normal renal function from a prior study [34]. Equation (2) was used to calculate the recommended dose for children with impaired renal function [24, 30].

2 $$\begin{aligned}&Recommended\;Dose\;\\&=\frac{Mean\;AUC_{\left(Healthy\right)}}{Mean\;AUC_{\left(Renal\;impaired\right)}}\times Stan dard\;Dose\end{aligned}$$

Pharmacodynamic target attainment was evaluated by assessing the % T > MIC in the pediatric populations with RI.

### Ethical approval

All physiological, pharmacokinetic, and demographic data used in this investigation retrieved from previously published articles and referenced appropriately. As no human subjects were involved in the current study, institutional ethical review was deemed unnecessary. Since each of the clinical trials’ individual investigations has been previously approved by the relevant ethical committees.

## Results

### PBPK model of cefotaxime in adults

**Fig. 1:**
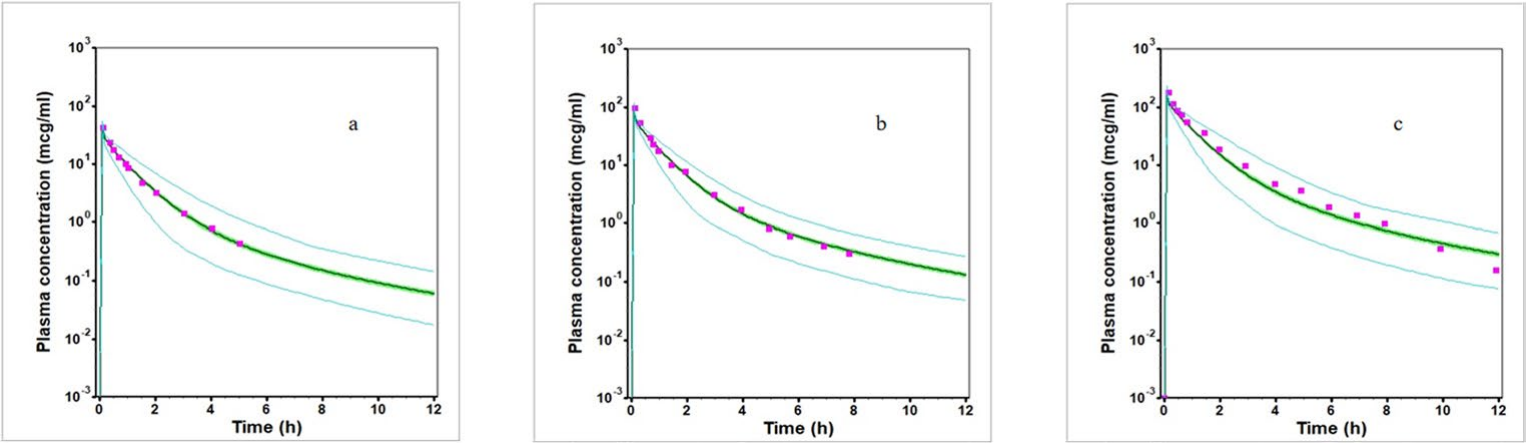
Observed (pink squares) and predicted (black line) Cp-time profiles of cefotaxime following intravenous infusion (500–2000 mg in 5 min) in healthy adults [31], (**a**) 500 mg, (**b**) 1000 mg, and (**c**) 2000 mg, with 90% confidence interval (light green) and 95th percentile (turquoise)

A PBPK model of CFT was developed and validated with GastroPlus^®^ using input parameters mentioned in Table S5. The initial model was defined in an adult with normal renal function from one clinical study [31]. Further evaluation of the model involved datasets from 8 studies. According to studies, CFT was primarily administered as IV infusion (2–20 min), with limited bolus dosing (Table S1). The mean plasma concentration–time profiles for CFT in adults as predicted by the model proved the validation of PBPK models. Visual predictive checks demonstrated that observed concentrations were contained within 90% confidence interval and 95th percentiles of simulated populations (Fig. 1; Figure S1). With increasing dose administration, the predicted and observed data (AUC_0−∞_ and C_max_) increased correspondingly. FE values were in the acceptable limit. Model was reasonably reproduced AUC_0−∞_, AUC_0−t_, and C_max_ with FE ranges between 0.5 and 2.0 (Table 1). The geometric mean fold error (GMFE) for AUC_0−∞_ and C_max_ were 1.103 and 1.181, respectively (Table S9).

**Table 1:** Observed versus physiological based pharmacokinetic (PBPK) model-derived pharmacokinetic parameters in adults without renal impairment

Dosages	Predicted AUC_0−∞_^a^ (µg.hr/mL)	Observed AUC_0−∞_ (µg.hr/mL)	FE^b^	Predicted AUC_0−t_^c^ (µg.hr/mL)	Observed AUC_0−t_ (µg.hr/mL)	FE	Predicted C_max_^d^ (µg/mL)	Observed C_max_ (µg/mL)	FE	Reference
500 mg, IV bolus	29.84	28	1.06	29.6	25.77	1.15	47.45	38.09	1.25	[9]
1000 mg, IV bolus	60.41	65.84	0.92	59.82	62.99	0.95	95.13	103.56	0.92	
2000 mg, IV bolus	123.64	119.27	1.04	122.43	115.24	1.06	190.96	202.66	0.94	
1000 mg, IV bolus	67.27	68.28	0.98	66.79	68.07	0.98	114.7	92.26	1.24	[25]
500 mg, IV bolus	30.47	25.57	1.19	30.18	24.25	1.24	48.87	35.16	1.38	[21]
1000 mg, IV bolus	61.69	60.33	1.02	61.1	59.64	1.02	97.95	78.06	1.25	
2000 mg, IV bolus	126.35	119.25	1.06	125.13	117.68	1.06	196.64	200	0.98	
2000 mg, IV 2 min	127.91	152.39	0.84	126.68	147.37	0.86	199.9	169	1.18	[15]
500 mg, IV bolus	30.98	32.04	0.97	30.69	30.81	0.99	58.07	62.89	0.92	[17]
1000 mg, IV bolus	62.76	55.65	1.12	62.16	54.76	1.14	116.32	67.76	1.71	
1000 mg, IV 20 min	65.89	53.75	1.22	65.25	52.78	1.23	67.18	57.37	1.17	[18]
1000 mg, IV 30 min	59.44	43.22	1.37	58.84	41.8	1.4	50.47	40.8	1.24	[14]
2000 mg, IV 30 min	130.07	142.53	0.91	128.79	141.9	0.91	114.08	124.4	0.92	[42]

^a^AUC_0−∞_: 0 to infinity hours blood drug concentration-area under the time curve, ^b^FE=fold error, ^c^AUC_0−t_: 0 to last sampling time hours blood drug concentration-area under the time curve, ^d^C_max_: Peak blood concentration

### PBPK model of cefotaxime in adults with renal impairment

**Fig. 2:**
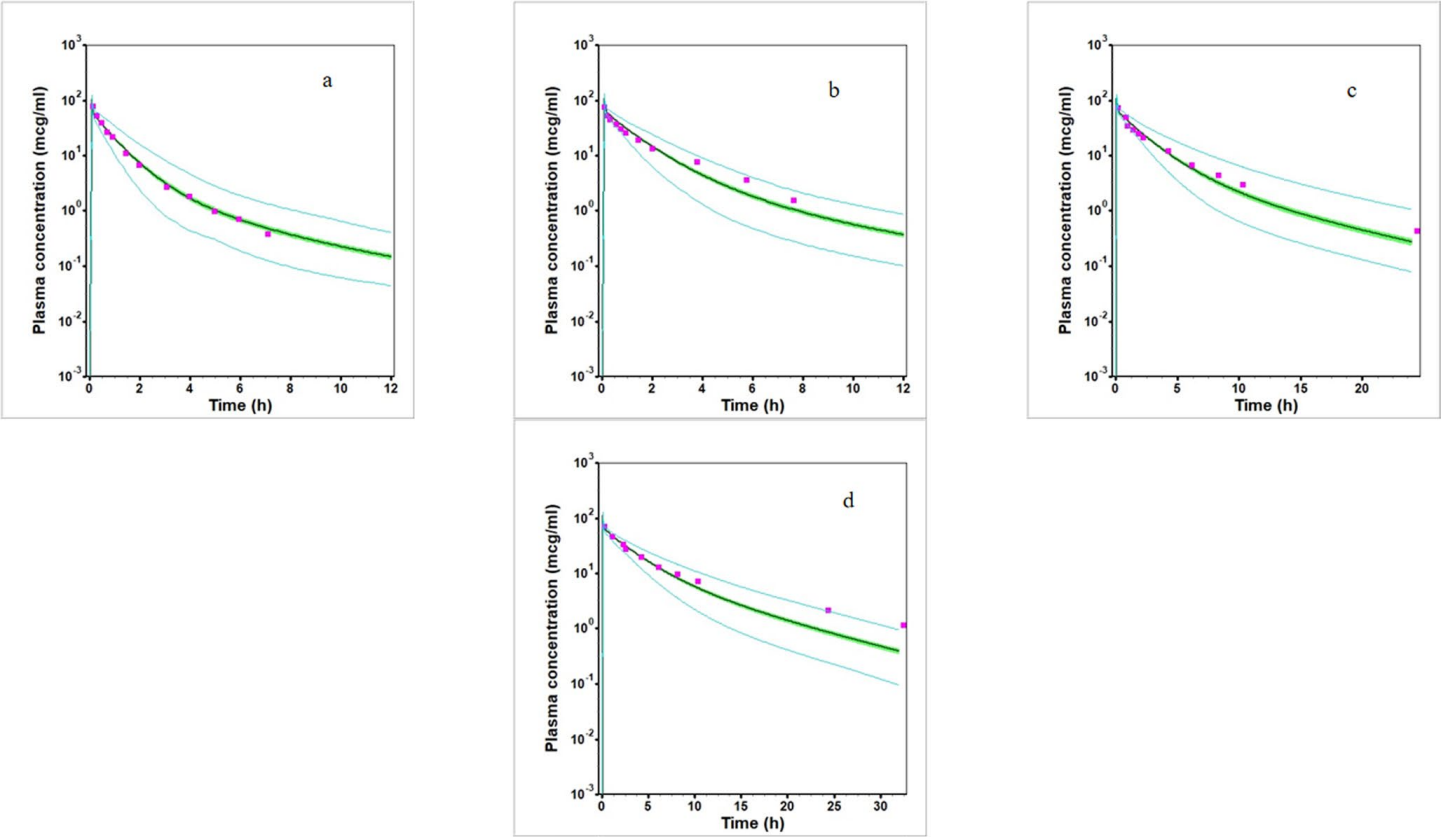
Observed (pink squares) and predicted (black line) Cp-time profiles of cefotaxime following intravenous administration (15 mg/kg body weight, bolus) in adults [22], (**a**) normal renal function, (**b**) moderate renal impairment, and (**c**) severe renal impairment, (**d**) end-stage renal disease, with 90% confidence interval (light green) and 95th percentile (turquoise)

The adult PBPK model was scaled to adults with RI, incorporating reductions in glomerular filtration rate and OAT3-mediated tubular secretion. The data set from one clinical study was used to calibrate the initial model to adults with RI [22]. In contrast, an independent data set from another study was employed to validate this model [33]. The mean plasma concentration–time profile for CFT administration was predicted and compared with the observed data from clinical studies [22, 33]. Evaluation of the model in adults with RI indicates that it described the pharmacokinetic profile of CFT in this patient populations. The population predicted plasma concentration-time profiles, including 90% CI and 95% percentile are shown in Fig. 2. Simulation demonstrated a progressive reduction in CFT clearance with increasing RI severity, resulting in 1.57-, 2.9-, and 4.5-fold higher AUC_0−∞_ in moderate, severe, and end-stage RI, respectively (Figure S2). Figure S3 also mentioned the comparison of predicted plasma concentration-time profiles with the observed data from the clinical studies [33]. The predicted AUC_0−∞_ and C_max_ were consistent with those observed in previous clinical studies with fold error ranges 0.87–1.12 and 0.86–1.62, respectively (Table 2). GMFE for AUC_0−∞_ were 1.009, 1.07, and 1.074 for moderate, severe, and end-stage renal impairment, respectively (Table S9).

**Table 2:** Observed verses physiologically-based pharmacokinetic model-derived pharmacokinetic parameters in adult population with renal impairment

Dosages	Renal impairment stage	Predicted AUC_0−∞_^a^ (µg.hr/mL)	Observed AUC_0−∞_ (µg.hr/mL)	FE^b^	Predicted AUC_0−t_^c^ (µg.hr/mL)	Observed AUC_0−t_ (µg.hr/mL)	FE	Predicted C_max_^d^ (µg/mL)	Observed C_max_ (µg/mL)	FE	Reference
15 mg/kg IV bolus	Moderate renal impairment	96.93	96.12	1.01	95.34	92.67	1.03	108.35	76.03	1.42	[22]
15 mg/kg IV bolus	Severe renal impairment	179.24	185.12	0.97	177.15	181.89	0.97	110.15	73.4	1.5	
15 mg/kg IV bolus	End-stage renal disease	278.42	321.23	0.87	274.79	306.73	0.89	110.1	69.85	1.57	
1000 mg, IV 5 min	Moderate renal impairment	91.72	90.73	1.01	90.22	84.53	1.07	102.5	63	1.62	[33]
1000 mg, IV 5 min	Severe renal impairment	154.2	137.45	1.12	147.29	129.71	1.14	94.21	107	0.88	
1000 mg, IV 5 min	End-stage renal disease	250.75	249.53	1.004	229.66	208.18	1.10	106.79	123.5	0.86	

^a^AUC_0−∞_: 0 to infinity hours blood drug concentration-area under the time curve, ^b^FE=fold error, ^c^AUC_0−t_: 0 to last sampling time hours blood drug concentration-area under the time curve, ^d^C_max_: Peak blood concentration

### PBPK model of cefotaxime in pediatrics

The adult PBPK model was scaled to the pediatric population with normal renal function by incorporating age-related physiological changes and scaling renal clearance parameters. OAT3 activity and GFR were optimized to match the observed data from one clinical study and validated utilizing data from another clinical study [23, 41]. Figure S4 mentioned the comparison of predicted plasma concentration-time profiles with the observed data. It proved the prediction accuracy of the model with fold error ranges in between 0.5 and 2, as mentioned in Table 3. GMFE for AUC_0−∞_ and C_max_ were 1.297 and 1.493, respectively (Table S9).

**Table 3:** Observed verses physiologically-based pharmacokinetic model-derived pharmacokinetic parameters in pediatric population with or without renal impairment

Dosages	Normal/Renal impairment stage	Predicted AUC_0−∞_^a^ (µg.hr/mL)	Observed AUC_0−∞_ (µg.hr/mL)	FE^b^	Predicted AUC_0−t_^c^ (µg.hr/mL)	Observed AUC_0−t_ (µg.hr/mL)	FE	Predicted C_max_^d^ (µg/mL)	Observed C_max_ (µg/mL)	FE	Reference
25 mg/kg IV 1 min^e^	Normal renal function	71.88	56.69	1.26	71.39	55.38	1.28	107	56.14	1.91	[23]
25 mg/kg IV 1 min^f^	Normal renal function	67.3	41.7	1.61	66.7	38.4	1.73	90.6	50.3	1.80	
50 mg/kg IV 30 min^g^	Normal renal function	164.13	217.7	0.75	162.66	215.64	0.75	170.7	132.4	1.28	[41]
50 mg/kg IV 20 min^h^	Normal renal function	223.3	233.91	0.95	220.92	223.27	0.98	209.3	185.2	1.13	[34]
50 mg/kg IV 20 min^i^	Moderate renal impairment	234.27	274.58	0.85	230.97	254.11	0.91	224.6	182.9	1.22	
50 mg/kg IV 20 min^j^	Severe renal impairment	430.81	481.44	0.89	417.66	453.08	0.92	245.98	228.8	1.07	

^a^AUC_0−∞_: 0 to infinity hours blood drug concentration-area under the time curve, ^b^FE=fold error, ^c^AUC_0−t_: 0 to last sampling time hours blood drug concentration-area under the time curve, ^d^C_max_: Peak blood concentration, ^e^7.7 months, ^f^7 years, ^g^1.8 years, ^h^13 years, ^i^11 years, ^j^13 years

### PBPK model of cefotaxime in pediatrics with renal impairment

**Fig. 3:**
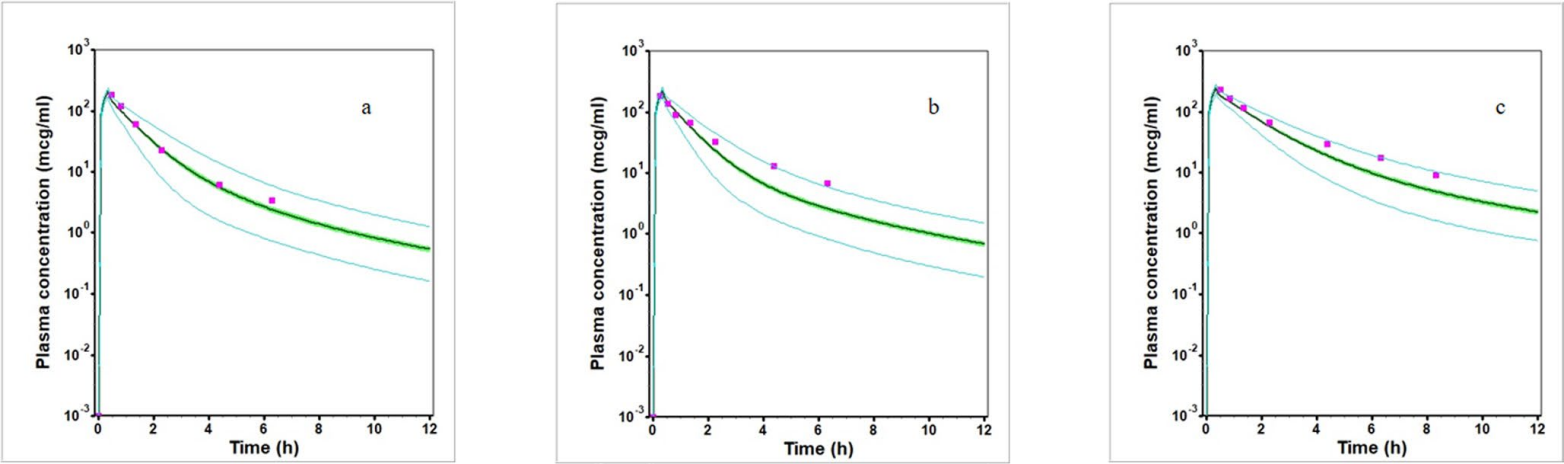
Observed (pink squares) and predicted (black line) Cp-time profiles of cefotaxime following intravenous infusion (50 mg/kg in 20 min), as simulated by the PBPK model in pediatrics [34], (**a**) normal renal function, (**b**) moderate renal impairment, and (**c**) severe renal impairment, with 90% confidence interval (light green) and 95th percentile (turquoise)

The pediatric model was further adapted to pediatric population with RI. For this purpose, the transporter activity (OAT3) and GFR were optimized to match the observed clinical data from children aged 7 to 16 years. Paap et al., [34] The scaled model reasonably reproduced CFT exposure in pediatrics with moderate and severe RI. Figure 3 displays the predicted and observed plasma-concentration profile in these populations, including the 90% confidence interval and 95th percentiles. The predicted PK data were accordant with the observed data [34]. with fold error values ranging from 0.85 to 0.89 for AUC_0−∞ and_ 1.07 to 1.22 for C_max_ (Table 3). An increase in AUC_0−∞_ was 1.05-fold in moderate RI and 1.93-fold in severe RI (Figure S5). PBPK model-derived pharmacokinetic parameters in pediatric population with or without renal impairment at a 50 mg/kg body weight dose level are mentioned in Table S10. Based on the increment in AUC_0−∞_, the dose was adjusted for pediatrics with renal impairment.

### Dosage adjustment in patients with renal impairment

We utilized developed PBPK models in adult and pediatric populations with impaired renal function to predict the outcome of renal impairment on CFT internal exposure and recommend the appropriate dosages for these populations interpreted with appropriate caution. AUC_0−∞_ served as a predictive indicator of CFT exposure in RI. Dosage optimization was performed using this parameter to meet the patient-specific features and age-specific target PK profiles.

In pediatric patients, model-informed dose reductions to 40%-73% of the standard adult dose are recommended, which achieved 52.5–77.5% T > MIC in these patients (Table 4). The recommended CFT dosages for pediatrics with moderate and severe RI were 35 mg/kg BW and 24 mg/kg BW, respectively (Table 4). At these doses, simulation predicted attainment of pharmacodynamic targets, with 56.25% and 84.5% T > MIC for moderate and severe RI, assuming an 8-hour dosing interval and MIC values of 1–4 mg/L, respectively. All of these findings show how useful PBPK modeling is for supporting CFT precision dosing in both adult and pediatric patients with RI.

**Table 4:** Dose recommendations utilizing the physiologically-based pharmacokinetic model-derived pharmacokinetic parameters in adult and pediatric population with renal impairment

Population	Normal/Renal impairment stage	Observed AUC_0−∞_^a^ (µg.hr/mL)	Predicted AUC_0−∞_ (µg.hr/mL)	Increment in AUC_0−∞_	Standard dose (mg/kg of BW)	Percent dose reduction (%)	Recommended dose (mg/kg of BW)	Predicted AUC_0−∞_ after dose adjustment (µg.hr/mL)	%T> MIC^b^
Adults^c^	Normal renal function	60.33	61.69	----	15	----	----	----	41.3
	Moderate renal impairment	96.12	96.93	1.57		40	9	57.48	52.5
	Severe renal impairment	185.12	179.24	2.9		60	6	70.74	65
	End-stage renal disease	321.23	278.42	4.5		73	4	74.24	77.5
Pediatrics^d^	Normal renal function	233.91	223.3	----	50	----	----	----	63.75
	Moderate renal impairment	274.58	234.27	1.05		30	35	219.79	56.25
	Severe renal impairment	481.44	430.81	1.93		52	24	265.21	84.5

^a^AUC_0−∞_: 0 to infinity hours blood drug concentration-area under the time curve, ^b^the percentage of time that elapses between subsequent antibiotic dosages while the antibiotic’s concentration remains higher than the minimum inhibitory concentration (MIC) i.e. 4 mg/L of cefotaxime, ^c^ [22], ^d^ [34]

## Discussion

PBPK modeling has become an integral component of drug research and regulatory science, used as a crucial tool in clinical-decision-making pharmaceutical development, and supervising evaluations of pediatric drug therapies [29]. Multiple modeling reports have been published and submitted to regulatory agencies, supporting requests for clinical research waivers or optimizing clinical trial designs. The use of PBPK modeling to establish initial dosages for pediatrics clinical trials has been more popular and accepted by regulators [44]. In this context, the present study developed the PBPK model, which reasonably well predicted CFT internal exposure in adult and pediatric participants with or without RI. This study provided valuable information to support pediatric dosing recommendations.

CFT is predominantly eliminated via kidney through a combination of glomerular filtration and active secretion [48], affected with impaired renal function, and causing its accumulation [22]. Additionally, children with low protein binding rates have lower levels of plasma proteins in RI, and more stringent dosage adjustments are required [33].

PBPK modeling has been effectively used in several studies to predict the PK of renally excreted drugs in pediatrics populations with RI. A researcher used PBPK modeling to characterize the PK of another drug in pediatrics with RI, incorporating glomerular filtration and active tubular secretion [49]. This approach aligns with ours, as the current model accounts for GFR, kidney size, and transporter activity when designing a representative patient population with RI.

Our published recommendations for PBPK modelling of OAT probes in RI were adopted to develop the validated CFT model for individuals with RI [36]. Our PBPK model confirmed a tendency of decreasing clearance of CFT and increasing its plasma exposure (AUC_0−∞_) with disease progression. As a result, CFT dosage administration in RI is recommended to be modified. Our proposal to lower OAT activity beyond the fall of GFR in moderate and severe RI was validated by the ability of the CFT model in patients with RI to accurately predict the RI-induced changes to CFT plasma exposure [36]. Therefore, the current study reinforces the role of PBPK modeling in supporting personalized-dosing strategies for drugs subjected to transporter-mediated elimination and predicting the outcome of RI on the pharmacokinetics of OAT-dependent active secretion.

Across adult and pediatric population, the PBPK simulations predict a progressive increase in CFT exposure with increasing severity of RI, supporting the dose reduction beyond empirical scaling. We made inferences based on variations in AUC between patients with or without RI. Adults should receive reduced doses in moderate (60% of standard CFT dose), severe (40% of standard CFT dose), and end-stage RI (27% of standard CFT dose). For children with mild RI, dose adjustment is not necessary. However, model results suggest the dosage should be changed to 60% and 48% of the standard pediatric dose for moderate and severe renal impairment, respectively. An important consideration is whether the recommended CFT dosage in patients with RI potentially achieves the pharmacodynamic attainment of 50–80% T > MIC. It was found that these dosages were able to attain 56.25–84.5% T > MIC in pediatrics with RI.

Although our study offers insightful information about CFT dosage in pediatric RI patients, it should be noted that it has certain limitations. Only a few research papers were found following a thorough literature review, which constrained the model’s evaluation due to the scarcity of pediatrics data. The safety and effectiveness of adjusted CFT regimens in children, especially those with RI, will need to be confirmed in the future through focused clinical pharmacokinetic studies. These studies must be supplemented by concurrent assessment of PK variability and age-related maturation of pharmacodynamic (PD) pathways that control pharmacological effects i.e., antibacterial activity. Future applications of this model may also incorporate inter-individual variability in disease progression to further validate and optimize it.

## Conclusion

The current study has demonstrated the first application of PBPK modeling to CFT dosage recommendations in a pediatric population with RI. The developed model was validated to reproduce CFT exposure in adults and pediatrics with or without RI by mechanistically accounting for changes in OAT transporter activity and GFR. The model-informed dose reduction was proposed for children with severe RI require a dose of 24 mg/kg BW, whereas those with moderate RI require 35 mg/kg BW. For all optimized CFT dosages, the 56.25–84.5%T > MIC has been attained in pediatric populations with RI. This PBPK framework supports rational, model-informed dosing of CFT in pediatric patients with varying renal impairment and supports dose recommendation development for high-risk populations.

## Supplementary information

Below is the link to the electronic supplementary material.

Supplementary File 1 (DOC 409 KB)

## Data Availability

Data will be available from the corresponding author on a sensible request from any researcher.
