# Supplementary material for: Physiologically based pharmacokinetic modeling of cefotaxime to inform pediatric dosing in renal impairment
Source: Eur J Clin Pharmacol. 2026 Jul 10;82(8):196. doi: 10.1007/s00228-026-04121-8 (PMC13350114; doi:10.1007/s00228-026-04121-8)
Supplement: Supplementary file 1 — (DOC 409 KB) [file 228_2026_4121_MOESM1_ESM.doc]

**Supplementary File #1**

**Title**

**Physiologically Based Pharmacokinetic Modeling of Cefotaxime to Inform Pediatric Dosing in Renal Impairment**

Najia Rahim ^a^*, Muhammad Sarfraz^b^, Muhammad Wahajuddin^c*^.

^a^Department of Pharmacy Practice, Dow College of Pharmacy, Dow University of Health Sciences, Karachi,

Pakistan.

^b^College of Pharmacy. Al Ain University, Al Ain, 64141, United Arab Emirates.

^c^School of Pharmacy & Medical Sciences, Faculty of Life Sciences, University of Bradford, UK.

***Corresponding authors**: (a) Najia Rahim (b) Muhammad Wahajuddin

**ORCID IDs**

Najia Rahim: 0000-0002-9874-8134

Muhammad Sarfraz: 0000-0002-0516-4966

Muhammad Wahajuddin: 0000-0003-3362-7558

**Note:** References in the following text are numbered based on the list in the main manuscript.

**Table S1.** Details of population characteristics from the clinical studies utilized in PBPK model development and validation.

| **Data sets** | **Dose** | **Number of subjects** | **Route** | **Age (years)** | **Weight (kg)** | **Reference** |
| --- | --- | --- | --- | --- | --- | --- |
| Model development | 500 mg | 6 | IV, 5 min | 25* | 69 (64- 73) | (Lüthy et al., 1981) |
|  | 1000 mg | 6 | IV, 5 min | 25* | 69 (64- 73) |  |
|  | 2000 mg | 6 | IV, 5 min | 25* | 69 (64- 73) |  |
| Model validation | 1000 mg | 10 | IV, 5 min | 32 (21-51) | 66.7 (44-82) | (Kemmerich et al., 1983) |
|  | 500 mg | 34 | IV bolus | 37 (20 - 55) | 75* | (Esmieu et al., 1980) |
|  | 1000 mg |  | IV, 5 min | 37 (20 - 55) | 75* |  |
|  | 2000 mg |  | IV, 5 min | 37 (20 - 55) | 75* |  |
|  | 500 mg | 3 | IV, 5 min | 30 (23 - 50) | 73 (60.5 - 82) | (R. Ings et al., 1985) |
|  | 1000 mg | 3 | IV, 5 min | 30 (23 - 50) | 73 (60.5 - 82) |  |
|  | 2000 mg | 3 | IV, 5 min | 30 (23 - 50) | 73 (60.5 - 82) |  |
|  | 500 mg | 4 | IV, bolus | 34 | 74 | (Harding et al., 1981) |
|  | 1000 mg | 4 | IV, bolus | 34 | 74 |  |
|  | 1000 mg | 8 | IV, 20 min | 54 ± 14^※^ | 63 ± 8 | (Hary et al., 1989) |
|  | 1000 mg | 10 | IV, 30 min | 26.4 (21-33) | 78.3±3.7 | (Fu et al., 1979) |
|  | 2000 mg |  | IV, 2 min | 29.8 (23 - 35) | 69.8 (50 - 83) | (Glöckner et al., 1980) |
|  | 2000 mg | 10 | IV, 30 min | 25 (20-36) | 65.4 (52.6-78) | (Vallee & LeBel, 1991) |

*Population averages were incorporated in the model, ^※^mean±standard deviation.

**Table S2.** Details of population characteristics from the clinical studies involving adult patients with renal impairment.

| **Data sets** | **Renal impairment stage** | **Dose** | **Route** | **Age**  **(years)** | **Weight**  **(kg)** | **Number of subjects** | **Reference** |
| --- | --- | --- | --- | --- | --- | --- | --- |
| Model development | Moderate renal impairment | 15 mg/kg | IV, bolus | 35  (22 - 77) | 75  (44.5 - 102) | 6 | (R. M. Ings et al., 1982) |
|  | Severe renal impairment |  |  |  |  | 6 |  |
|  | End-stage renal disease |  |  |  |  | 6 |  |
| Model validation | Moderate renal impairment | 1000 mg | IV, 5 min | 39.6 ± 14.9^※^ | 68.4 ± 10.4 | 8 | (Matzke et al., 1985) |
|  | Severe renal impairment |  |  | 45.6 ± 15.4 | 82.5 ± 13.8 | 8 |  |
|  | End-stage renal disease |  |  | 50.0 ± 15.2 | 66.3 ± 9.3 | 8 |  |

^※^mean±standard deviation.

**Table S3.** Details of population characteristics from the clinical studies involving pediatric patients with or without renal impairment.

| **Data sets** | **Normal/Renal impairment stage** | **Dose** | **Route** | **Age**  **(years)** | **Body weight**  **(kg)** | **Number of subjects** | **Reference** |
| --- | --- | --- | --- | --- | --- | --- | --- |
| Model development | Normal renal function | 25 mg/kg | IV, 1-2 min | 0.58 (0.4-0.9) | 8.5* | 5 | (Kafetzis et al., 1981) |
|  |  |  |  | 0.58 (2-12) | 23* | 21 |  |
| Model validation | Normal renal function | 50 mg/kg | IV, 30 min | 1.75±3.17^※^ | 11.7±8.9 | 13 | (Trang et al., 1985) |
|  | Normal renal function |  | IV, 20 min | 13 (7-16) | 44.23±18.34 | 7 | (Paap et al., 1991) |
|  | Moderate  renal impairment |  | IV, 20 min | 11 (7-16) | 34.13±12.74 | 6 |  |
|  | Severe  renal impairment |  | IV, 20 min | 13 (7-16) | 39.07±12.19 | 6 |  |

*Population averages were used incorporated in the model, ^※^mean±standard deviation.

**Table S4.** Physiological parameters utilized in the cefotaxime adult PBPK model.

| **Tissue^a^** | **Perfusion**  **(mL/sec)** | **Volume**  **(mL)** | **Kp^b^** |
| --- | --- | --- | --- |
| Hepatic Artery | 9.144 | 0 | 0 |
| Arterial Supply | 93.212 | 2100.68 | 0 |
| Venous Return | 93.212 | 4201.36 | 0 |
| ACAT Gut | 11.267 | 0 | 0 |
| Lung | 6.7998 | 1063.91 | 0.68 |
| Adipose | 6.7998 | 20419.89 | 0.62 |
| Muscle | 11.877 | 23754.12 | 0.63 |
| Liver | 23.238 | 1517.84 | 0.63 |
| Spleen | 2.8273 | 169.63 | 0.64 |
| Heart | 3.8286 | 314.67 | 0.66 |
| Brain | 12.692 | 1493.16 | 0.62 |
| Kidney | 21.195 | 345.57 | 0.65 |
| Skin | 5.4101 | 2705.07 | 0.71 |
| ReproOrg | 0.1628 | 46.51 | 0.66 |
| RedMarrow | 5.3029 | 1060.58 | 0.66 |
| YellowMarrow | 1.474 | 2948.05 | 0.62 |
| RestOfBody | 1.2317 | 2463.3 | 0.65 |

^a^All tissues were set as permeability limited, ^b^Kps were calculated using Poullin- Theil extracellular equation.

**Table S5.** Input parameters used in the development of initial physiological based pharmacokinetic (PBPK) cefotaxime model.

| **Parameter** | **Literature values** | **Optimized values used in initial PBPK model** |
| --- | --- | --- |
| Molecular weight | 455.47 g/mol^a^ | ---- |
| logP | -0.63^a^ | ---- |
| pKa (acid) | 2.51, 71.77^a^ | ---- |
| pKa (base) | 3.98, .71.77^a^ | ---- |
| Water solubility | 1.75 g/ml at pH 3.41^a^ | ---- |
| Fup | 42.26%^a^ | 60%^b^ |
| Blood-to-plasma ratio | 0.93^a^ | ---- |
| **OAT3** | | |
| V_max_ | 305 ±28 nmol*(mg protein)(-1) /min^c^ | 0.62  mg/sec/mg. protein |
| K_m_ | 326.6  mg/L^c^ | 326.6  mg/L |
| Distribution | Permeability-limited method | Specific PStc (initial value=0.1, optimized value= 0.02608, final value=0.03) |
| Hepatic metabolism | 4 L/hr  (25% of Cl_total_)^d^ | ---- |
| Renal clearance via passive transport | 1.814 L/hr (10% of Cl_total_)^d^ | ---- |

^a^ADMET predictor values, ^b^, ^c^(Yee, et al.,2013), ^d^(Todd PA, Brogden RN., 1990).

**Table S6**. Scaling of the adult PBPK model of cefotaxime for adult patients with renal impairment (R. M. Ings et al., 1982).

| **Normal/Renal impairment stage** | **Cl_cr_^a^**  **from clinical study**  **(ml/min)** | **Cl_total_^b^**  **from clinical study**  **(ml/min)** | **Cl_renal_^c^ from clinical study**  **(ml/min)** | **Cl_nonrenal_ from clinical study**  **(ml/min)** | **Reduction in Cl_renal_**  **from clinical study**  **(%)** | **Reduction in**  **Cl_nonrenal_ from clinical study**  **(%)** | **GFR^d^ input in the adult PBPK model**  **(ml/min)** | **OAT3^e^ V_max_ input in the adult PBPK model**  **(mg/sec/mg. protein)** | **Cl_nonrenal_ input in the adult PBPK model**  **(ml/min)** | **Scaling factor for Cl_reanal_ used in the adult PBPK model** | **Scaling factor for Cl_nonreanal_ used in the adult PBPK model** |
| --- | --- | --- | --- | --- | --- | --- | --- | --- | --- | --- | --- |
| Normal renal function | 143.44 | 355 | 116 | 239 | 0 | 0 | 30 | 0.62 | 66.67 | 1 | 1 |
| Moderate renal impairment | 49.93 | 187.7 | 50.8 | 138 | 43.8 | 57.7 | 18 | 0.37 | 53.33 | 0.6 | 0.8 |
| Severe renal impairment | 18.35 | 161 | 23.5 | 137 | 20.3 | 57.3 | 6 | 0.124 | 53.33 | 0.2 | 0.8 |
| End-stage renal disease | 5.35 | 149 | 5.3 | 144 | 4.6 | 60.3 | 3 | 0.062 | 40 | 0.1 | 0.6 |

^a^Creatinine clearance, ^b^Total clearance, ^c^Renal clearance, ^d^Glomerular filtration rate, ^e^OAT3 transporter at the baso-lateral membrane of renal proximal tubule cells of kidney, SF calculated by using the equation mentioned below.


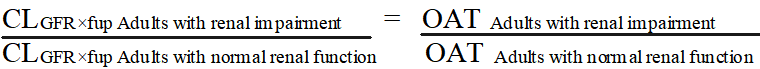


**Table S7.** Scaling of the adult PBPK model of cefotaxime for pediatrics without renal impairment (Kafetzis et al., 1981).

| **Age group for the virtual population used in the PBPK model**  **(years)** | **Age of virtual subject used in the PBPK model**  **(years)** | **Cl_total_**  **from clinical study**  **(ml/min)** | **Cl_nonrenal_^b^**  **from clinical study**  **(ml/min)** | **Cl_renal_^c^**  **from clinical study**  **(ml/min)** | **Reduction in Cl_renal_**  **from clinical study**  **(%)** | **Reduction in**  **Cl_nonrenal_**  **from clinical study**  **(%)** | **GFR^d^ input in the pediatric PBPK model**  **(ml/min)** | **OAT3^e^ V_max_**  **input in the pediatric PBPK model**  **(mg/sec/mg. protein)** | **Cl_nonrenal_ input in the pediatric PBPK model**  **(ml/min)** | **Scaling factor for Cl_reanal_ used in the pediatric PBPK model** | **Scaling factor for Cl_nonreanal_ used in the pediatric PBPK model** |
| --- | --- | --- | --- | --- | --- | --- | --- | --- | --- | --- | --- |
| 25-55^f^ | 40 | 326 | 164 | 162 | 0 | 0 | 30 | 0.62 | 66.67 | 1 | 1 |
| 0.25-1 | 0.7 | 58.65 | 62.4 | 60 | 37 | 38 | 6.67 | 0.248 | 26.67 | 0.4 | 0.4 |
| 1.1-2 | 1.5 | NA^g^ | NA | 33.93 | 20.94 | NA | 15 | 0.31 | 40 | 0.5 | 0.6 |
| 2.1-5 | 3.5 | NA | NA | NA | NA | NA | 21.23 | 0.44 | 53.33 | 0.7 | 0.8 |
| 5.1-11 | 7 | 239 | 103.5 | 136 | 83.9 | 63 | 24.22 | 0.48 | 60 | 0.8 | 0.9 |
| 11.1-16 | 13.5 | 122.4 | 62.4 | 60 | 37 | 38 | 27 | 0.558 | 60 | 0.9 | 0.9 |

^a^Creatinine clearance, ^b^Nonrenal clearance, ^c^Renal clearance, ^d^Glomerular filtration rate, ^e^OAT3 transporter at the basolateral membrane of renal proximal tubule cells of kidney, ^f^Data for adults from the clinical study by Luthy, et al. 1981, ^g^Not mentioned in the clinical study, SF calculated by using the equation mentioned below.


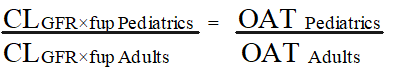


**Table S8.** Scaling of the pediatric PBPK model of cefotaxime for pediatric patients with renal impairment.

| **Pediatric group**  **(age, body weight)** | **Normal/Renal impairment stage** | **Cl_cr_^a^ from clinical study**  **(ml/min)** | **Cl_total_**  **from clinical study**  **(ml/min)** | **Cl_nonrenal_^b^**  **from clinical study**  **(ml/min)** | **Cl_renal_^c^**  **from clinical study**  **(ml/min)** | **Reduction in Cl_renal_**  **from clinical study**  **(%)** | **Reduction in**  **Cl_nonrenal_**  **from clinical study**  **(%)** | **GFR^d^ input in the pediatric PBPK model**  **(ml/min)** | **OAT3^e^ V_max_**  **input in the pediatric PBPK model**  **(mg/sec/mg. protein)** | **Cl_nonrenal_ input in the pediatric PBPK model**  **(ml/min)** | **Scaling factor for Cl_reanal_ used in the pediatric PBPK model** | **Scaling factor for Cl_nonreanal_ used in the pediatric PBPK model** |
| --- | --- | --- | --- | --- | --- | --- | --- | --- | --- | --- | --- | --- |
| 7 months, 8.4 kg | Normal renal function^f^ | 42 | 57.96 | 26.06 | 31.92 | 0 | 0 | 6.67 | 0.248 | 26.67 | 1 | 1 |
|  | Moderate renal impairment | NA^g^ | NA | NA | NA | NA | NA | 4 | 0.149 | 21.34 | 0.6 | 0.8 |
|  | Severe renal impairment | NA | NA | NA | NA | NA | NA | 1.33 | 0.049 | 21.34 | 0.2 | 0.8 |
|  | End-stage renal disease | NA | NA | NA | NA | NA | NA | 0.67 | 0.025 | 16 | 0.1 | 0.6 |
| 7 years, 23 kg | Normal renal function^f^ | 75 | 239.2 | 103.5 | 135.7 | 0 | 0 | 24.22 | 0.48 | 60 | 1 | 1 |
|  | Moderate renal impairment | NA | NA | NA | NA | NA | NA | 14.53 | 0.288 | 48 | 0.6 | 0.8 |
|  | Severe renal impairment | NA | NA | NA | NA | NA | NA | 4.84 | 0.096 | 48 | 0.2 | 0.8 |
|  | End-stage renal disease | NA | NA | NA | NA | NA | NA | 2.42 | 0.048 | 36 | 0.1 | 0.6 |
| 13 years, 39 kg | Normal renal function^h^ | 97.2 | 122.44 | 62.4 | 60.42 | 0 | 0 | 27 | 0.558 | 60 | 1 | 1 |
|  | Moderate renal impairment | 37.2 | 76.6 | 49.8 | 26.76 | 44.3 | 79.8 | 16.2 | 0.335 | 48 | 0.6 | 0.8 |
|  | Severe renal impairment | 12.26 | 58.8 | 50.86 | 7.9 | 13.1 | 81.5 | 5.4 | 0.1116 | 48 | 0.2 | 0.8 |
|  | End-stage renal disease | NA | NA | NA | NA | NA | NA | 2.7 | 0.0558 | 36 | 0.1 | 0.6 |

^a^Creatinine clearance, ^b^Nonrenal clearance, ^c^Renal clearance, ^d^Glomerular filtration rate, ^e^OAT3 transporter at the basolateral membrane of renal proximal tubule cells of kidney, ^f^Kafetzis, et al. 1981, ^g^Not available from reported literature, ^h^Paaps, et al 1991, SF calculated by using the equation mentioned below.


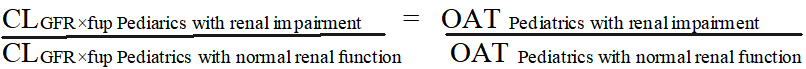


**Table S9.** Comparison PK parameters of cefotaxime PBPK models in adult and pediatrics.

| **Population** | **Normal/Renal impairment stage** | **PK parameter^a^** | **MFE^b^** | **GMFE^c^** |
| --- | --- | --- | --- | --- |
| Adult | Normal renal function | AUC_0-∞_ (µg.hr/mL) | 1.03375 | 1.103 |
|  |  | C_max_ (µg/mL) | 1.15 | 1.181 |
|  | Moderate  renal impairment | AUC_0-∞_ (µg.hr/mL) | 1.01 | 1.009 |
|  |  | C_max_ (µg/mL) | 1.52 | 1.515 |
|  | Severe  renal impairment | AUC_0-∞_ (µg.hr/mL) | 1.045 | 1.07 |
|  |  | C_max_ (µg/mL) | 1.19 | 1.304 |
|  | End-stage renal disease | AUC_0-∞_ (µg.hr/mL) | 0.937 | 1.074 |
|  |  | C_max_ (µg/mL) | 1.215 | 1.348 |
| Pediatrics | Normal renal function | AUC_0-∞_ (µg.hr/mL) | 1.1425 | 1.297 |
|  |  | C_max_ (µg/mL) | 1.53 | 1.493 |

^a^AUC_0-∞_: 0 to infinity hours blood drug concentration-area under the time curve; C_max_: Peak blood concentration; ^b^Mean fold error (MFE):$MFE=\frac{1}{n}\times\frac{\text{predicted}}{\text{observed}}$, ^c^Geometric mean fold error (GMFE):

**Table S10.** Physiologically-based pharmacokinetic model-derived pharmacokinetic parameters in pediatric population with or without renal impairment at 50 mg/kg body weight dose level.

| **Pediatric group**  **(age, body weight)** | **Normal/Renal impairment stage** | **Predicted AUC_0-∞_^a^ (µg.hr/mL)** | **Increment**  **in AUC_0-∞_** | **Predicted AUC_0-t_^b^ (µg.hr/mL)** | **Increment**  **in AUC_0-t_** | **Predicted C_max_^c^**  **(µg/mL)** | **Increment**  **in C_max_** |
| --- | --- | --- | --- | --- | --- | --- | --- |
| 7 months, 8.4 kg | Normal renal function | 145.57 | ---- | 144.58 | ---- | 219.64 | ---- |
|  | Moderate renal impairment | 244.76 | 1.68 | 241.26 | 1.67 | 244.5 | 1.11 |
|  | Severe renal impairment | 310.67 | 2.13 | 304.84 | 2.11 | 252.42 | 1.15 |
|  | End-stage renal disease | 431.54 | 2.96 | 416.66 | 2.88 | 260.12 | 1.18 |
| 8 years, 23 kg | Normal renal function | 183.11 | ---- | 181.53 | ---- | 241.88 | ---- |
|  | Moderate renal impairment | 232.55 | 1.27 | 229.72 | 1.26 | 250.85 | 1.04 |
|  | Severe renal impairment | 338.76 | 1.85 | 331.92 | 1.83 | 263.66 | 1.09 |
|  | End-stage renal disease | 484.71 | 2.65 | 467.68 | 2.58 | 271.32 | 1.12 |
| 13 years, 39 kg | Normal renal function | 223.27 | ---- | 220.92 | ---- | 209.3 | ---- |
|  | Moderate renal impairment | 319.48 | 1.43 | 314.09 | 1.42 | 247.56 | 1.18 |
|  | Severe renal impairment | 544.66 | 2.43 | 524.58 | 2.37 | 263.7 | 1.26 |
|  | End-stage renal disease | 812.7 | 3.64 | 749.91 | 3.39 | 270.14 | 1.29 |

^a^AUC_0-∞_: 0 to infinity hours blood drug concentration-area under the time curve, ^b^AUC_0-t_: 0 to last sampling time hours blood drug concentration-area under the time curve, ^c^C_max_: Peak blood concentration.

**Fig. S1(a)** shows the Cp-time profiles of cefotaxime following intravenous administration (500-2000 mg) in adults, as simulated by the PBPK model, open squares are observed values and green lines are predicted values for cefotaxime.

**Fig. S1(b)** shows the Cp-time profiles of cefotaxime following intravenous administration (500-2000 mg) in adults, as simulated by the PBPK model, open squares are observed values and green lines are predicted values for cefotaxime.

**Fig. S1(c)** shows the Cp-time profiles of cefotaxime following intravenous administration (500-2000 mg) in adults, as simulated by the PBPK model, open squares are observed values and green lines are predicted values for cefotaxime.

**Fig. S2** shows observed and predicted AUC0-α following intravenous administration of cefotaxime (15 mg/kg body weight, bolus) in adults with normal renal function, moderate renal impairment, severe renal impairment, and end-stage renal disease (R. M. Ings et al., 1982).

**Fig. S3** shows the Cp-time profiles of cefotaxime following intravenous administration (1000 mg) in adult population with impaired renal function, as simulated by the PBPK model, open squares are observed values and green lines are predicted values for cefotaxime.

**Fig. S4** shows the Cp-time profiles of cefotaxime following intravenous infusion (25 and 50 mg/kg body weight) in pediatric population with normal renal function, as simulated by the PBPK model, open squares are observed values and green lines are predicted values for cefotaxime.

**Fig. S5** shows observed and predicted AUC0-α following intravenous administration of cefotaxime (50 mg/kg in 20 min) in pediatric patients with normal renal function, moderate renal impairment, and severe renal impairment (Paap et al., 1991).
